# Spatially Defined InsP_3_-Mediated Signaling in Embryonic Stem Cell-Derived Cardiomyocytes

**DOI:** 10.1371/journal.pone.0083715

**Published:** 2014-01-07

**Authors:** Nidhi Kapoor, Joshua T. Maxwell, Gregory A. Mignery, David Will, Lothar A. Blatter, Kathrin Banach

**Affiliations:** 1 Heart Institute, Cedars-Sinai Medical Center, Los Angeles, California, United States of America; 2 Department of Cell and Molecular Physiology, Stritch School of Medicine, Loyola University Chicago, Maywood, Illinois, United States of America; 3 Department of Molecular Biophysics and Physiology, Rush University Medical Center, Chicago, Illinois, United States of America; 4 Center for Cardiovascular Research, Dept. of Medicine, Section of Cardiology, University of Illinois at Chicago, Chicago, Illinois, United States of America; Northwestern University, United States of America

## Abstract

The functional role of inositol 1,4,5-trisphosphate (InsP_3_) signaling in cardiomyocytes is not entirely understood but it was linked to an increased propensity for triggered activity. The aim of this study was to determine how InsP_3_ receptors can translate Ca^2+^ release into a depolarization of the plasma membrane and consequently arrhythmic activity. We used embryonic stem cell-derived cardiomyocytes (ESdCs) as a model system since their spontaneous electrical activity depends on InsP_3_-mediated Ca^2+^ release. [InsP_3_]_i_ was monitored with the FRET-based InsP_3_-biosensor FIRE-1 (Fluorescent InsP_3_ Responsive Element) and heterogeneity in sub-cellular [InsP_3_]_i_ was achieved by targeted expression of FIRE-1 in the nucleus (FIRE-1nuc) or expression of InsP_3_ 5-phosphatase (m43) localized to the plasma membrane. Spontaneous activity of ESdCs was monitored simultaneously as cytosolic Ca^2+^ transients (Fluo-4/AM) and action potentials (current clamp). During diastole, the diastolic depolarization was paralleled by an increase of [Ca^2+^]_i_ and spontaneous activity was modulated by [InsP_3_]_i_. A 3.7% and 1.7% increase of FIRE-1 FRET ratio and 3.0 and 1.5 fold increase in beating frequency was recorded upon stimulation with endothelin-1 (ET-1, 100 nmol/L) or phenylephrine (PE, 10 µmol/L), respectively. Buffering of InsP_3_ by FIRE-1nuc had no effect on the basal frequency while attenuation of InsP_3_ signaling throughout the cell (FIRE-1), or at the plasma membrane (m43) resulted in a 53.7% and 54.0% decrease in beating frequency. In m43 expressing cells the response to ET-1 was completely suppressed. Ca^2+^ released from InsP_3_Rs is more effective than Ca^2+^ released from RyRs to enhance I_NCX_. The results support the hypothesis that in ESdCs InsP_3_Rs form a functional signaling domain with NCX that translates Ca^2+^ release efficiently into a depolarization of the membrane potential.

## Introduction

In cardiac muscle the expression of inositol-1,4,5-triphosphate receptors (InsP_3_R) is most abundant during early development [1], [2]. In embryonic as well as neonatal cardiomyocytes the presence of all three InsP_3_R isoforms has been documented with the most prominent appearance of InsP_3_R1 and InsP_3_R2 [3], [4]. At embryonic and neonatal stages of differentiation, immunostainings indicate that InsP_3_Rs pre-dominantly locate to the nuclear envelope [4]–[6]. Receptor mediated G_q_-protein stimulation of these cells results in InsP_3_ production and concomitantly Ca^2+^ release events that occurred mainly at the nuclear envelope [4], [7], [8]. The functional role of InsP_3_Rs in the developing myocytes is not well understood, but in the embryonic heart tube, mouse and human embryonic stem cell-derived cardiomyocytes and human iPS cell-derived cardiomyocytes a role of InsP_3_R-mediated Ca^2+^ release in the generation of spontaneous electrical activity has been demonstrated [9]–[12].

In contrast to the abundance of InsP_3_Rs in the early developmental stages, their expression decreases towards adulthood; However, in the adult atrial [13] and ventricular muscle of rat [14], cat [15], and rabbit [16] the expression of InsP_3_R2 isoforms was demonstrated. In atrial myocytes its distribution is homogeneous throughout the cell, whereas in ventricular myocytes a prevalence in the nuclear envelope (rat) [14] and the dyadic junctions (mouse) [17] was reported. During excitation-contraction coupling in the adult cardiac muscle, Ca^2+^ is released from the sarcoplasmic reticulum mainly through the ryanodine receptor type 2 (RyR2), which is expressed 50 fold higher than InsP_3_Rs. In contrast InsP_3_R-mediated signaling has been linked to excitation-transcription coupling. Activation of nuclear InsP_3_Rs was sufficient for the activation and translocation of the transcription factor HDAC that remained unresponsive to beat-to-beat changes in [Ca^2+^]_i_ [18]. Nevertheless, despite the comparably low expression levels, InsP_3_Rs play a role in the induction of cardiac arrhythmia. Stimulation of InsP_3_R-mediated Ca^2+^ release results in increased spark frequency, positive inotropy, and an increase in arrhythmic spontaneous activity in atrial and ventricular myocytes [15], [16], [18]–[21]. As indicated by these studies, the amount of InsP_3_-mediated Ca^2+^ release appears low and may be more relevant as a facilitator of Ca^2+^ release from RyRs thus contributing indirectly to excitation-contraction coupling.

The sub-cellular location of InsP_3_-mediated Ca^2+^ release could critically influence its function. Whereas sub-sarcolemmal Ca^2+^ release can depolarize the membrane by activation of sodium calcium exchange (NCX), Ca^2+^ released at the nuclear envelope might have a higher likelihood to be removed by SERCA [19], [21]. The functional differences between spatially distinct Ca^2+^ signaling events are very pronounced in ESdCs. Localized Ca^2+^ release events through RyRs (sparks) can be frequently monitored throughout the ESdC, whereas localized release events through InsP_3_Rs (puffs) are seldom identified [8], [9]. Nonetheless, sparks are insufficient to maintain spontaneous activity, whereas InsP_3_ mediated Ca^2+^ release can sustain spontaneous activity even after depletion of the RyR operated Ca^2+^ stores or in RyR2 deficient ESdCs [9], [22].

We used ESdCs as a model to test the hypothesis that InsP_3_Rs close to the plasma membrane form functional signaling domains with NCX and that, in contrast to cytoplasmic or nuclear InsP_3_Rs, their Ca^2+^ release can be efficiently translated into I_NCX_ and a depolarization of the membrane potential (V_m_). For this purpose we determined the effect **i.** of InsP_3_R-mediated release on I_NCX_ and **ii.** of spatial inhomogeneities in InsP_3_ concentration on spontaneous activity [20].

## Materials and Methods

The culture of mouse embryonic stem cells (mES) of the cell line CMV (Specialty Media; Phillipsburg, NJ, USA), their differentiation into cardiomyocytes and use for laser scanning confocal microscopy are described in detail elsewhere [9], [23].

### FIRE-1 construct

As previously described [24] the FIRE-1 InsP_3_ biosensor was assembled using the InsP_3_R ligand-binding domain terminally fused with enhanced CFP and YFP at the amino and carboxyl termini, respectively. In FIRE-1 transfected COS-1 cells, rat neonatal, adult cat ventricular myocytes and ESdCs (Data S1) FIRE-1 exhibited comparable dynamic range and a 10% increase in donor (CFP) fluorescence upon bleaching of YFP, indicative of FRET [24].

### FIRE-1nuc construction

The FIRE-1 indicator was targeted to the nucleus by insertion of a triplet tandem of the SV40 large T-antigen nls using the following oligonucleotides: (sense: GGCTCGAGATCCAAAAAAGAAGAGAAAGGTAGATCCAAAAAAGAAGAGAAAGGTAGATCCAAAAAAGAAGAGAAAGGTATCTCGAAGG and antisense: CCCTCGAGATACCTTTCTCTTCTTTTTTGGATCTACCTTTCTCTTCTTTTTTGGATCTACCTTTCTCTTCTTTTTTGGATCTCGAGCC). The oligonucleotides were mixed and annealed by incubation at 90°C for 5 minutes and then cooling to room temperature, followed by digestion with Xho I and ligation into similarly digested FIRE-1 plasmid [24]. Expression and nuclear localization were verified in transiently transfected COS-1 cells by Western blotting with an IP_3_R1 specific amino-terminal antibody (T1NH; data not shown) and direct visualization with fluorescence microscopy. The insert harboring the FIRE-1nuc coding region was excised with Bgl II and ligated into Bgl II digested pShuttle-CMV vector (Stratagene; La Jolla, CA) for adenoviral production.

### m43 construction

The coding region of the mouse 43 kDa inositol polyphosphate 5′-phosphatase (m43) with N-terminal FLAG-tag from pcDNA3 (kindly provided by Dr. Elizabeth A. Woodcock, Baker Heart Research Institute, Melbourne, Victoria, Australia) was amplified by PCR using the following primers (sense: CGGGTCGACCCACCATGGACTACAAGGACGAC and antisense: GCCGTCGACTCACTGCACGACACAACA). The PCR product was digested with Sal I, ligated into similarly digested pCMV-5 vector, and expression was verified in COS-1 cells by transient transfection and Western blotting with anti-FLAG antibody (Affinity BioReagents) (Figure S2). The FLAG-tagged m43 coding region was excised with Sal I and ligated into Sal I digested pShuttle-CMV vector (Stratagene; La Jolla, CA) for adenoviral production.

### FIRE-1nuc and m43 adenovirus production

The adenoviruses were created using the commercially available AdEasyTM XL adenoviral vector system kit (Stratagene; La Jolla, CA). Briefly, the bacterial cell line BJ5183-AD-1, pre-transformed with the plasmid pAdEasy-1 was used for *in vivo* homologous recombination with either pShuttle-CMV-m43 or pShuttle-CMV-FIRE-1nuc. The pAdEasy-1-m43 or pAdEasy-1-FIRE-1nuc insert containing plasmids were separately transformed into DH5α and produced in bulk. Purified pAdEasy-1-m43 or pAdEasy-1-FIRE-1nuc was used to transfect AD-293 cells for virus amplification. Both viruses were plaque-purified, amplified, CsCl gradient-purified, and stored at −80°C.

### Adenoviral transduction and FRET measurements

24 hours post plating, dissociated ESdCs were transduced with recombinant replication-deficient adenovirus carrying sequence for either the InsP_3_ biosensor FIRE-1 [24], FIRE-1nuc (FIRE-1 sequence plus 3 tandem nuclear localization signals (3 tandem-DPKKKRKV)), or FLAG tagged m43 phosphatase [25]. After overnight incubation at a multiplicity of infection (MOI) of 1–10 the media was replaced. Changes in fluorescence resonance energy transfer (FRET) between the cyan fluorescent protein (CFP) and the yellow fluorescent protein (YFP) were measured by laser scanning confocal microscopy. CFP was excited with a 440 nm diode laser. CFP and YFP emissions were measured at 488 (*F*_488_) and >560 nm (*F*_560_), respectively. Changes in InsP_3_ activity are defined as the relative change in the background corrected ratio of *F*_560_/*F*_488_. To obtain a reliable reproducible readout for the changes induced by the pharmacological agents, the fluorescence was determined after 3 min of superfusion. The change was then quantified as the average fluorescence over the time period of 5 min. The experiments were conducted at room temperature. FRET between CFP and YFP was confirmed by photobleaching of the accepter molecule (YFP; Figure S1).

### Chemicals and statistics

Endothelin-1 (ET-1), phenylephrine (PE), and caffeine were diluted in H_2_O, 2-aminoethoxydiphenyl borate (2-APB), U73122 and U73343 were dissolved in dimethylsulphoxide (DMSO) and further diluted >1,000 fold for experiments. All chemicals were purchased from Sigma. Results are presented as mean ± SEM and *n* represents the number of experiments. Statistical differences between two groups were analyzed by student's t-test and considered significant at *P*<0.05. Multiple comparisons were performed by analysis of variance (ANOVA) and significant differences between the groups were identified with the *Tukey HSD Test* indicating significance at *P*<0.05. A detailed description of the confocal imaging, electrophysiological recordings, and immunocytochemistry can be found in Data S1.

## Results

**Figure 1:**
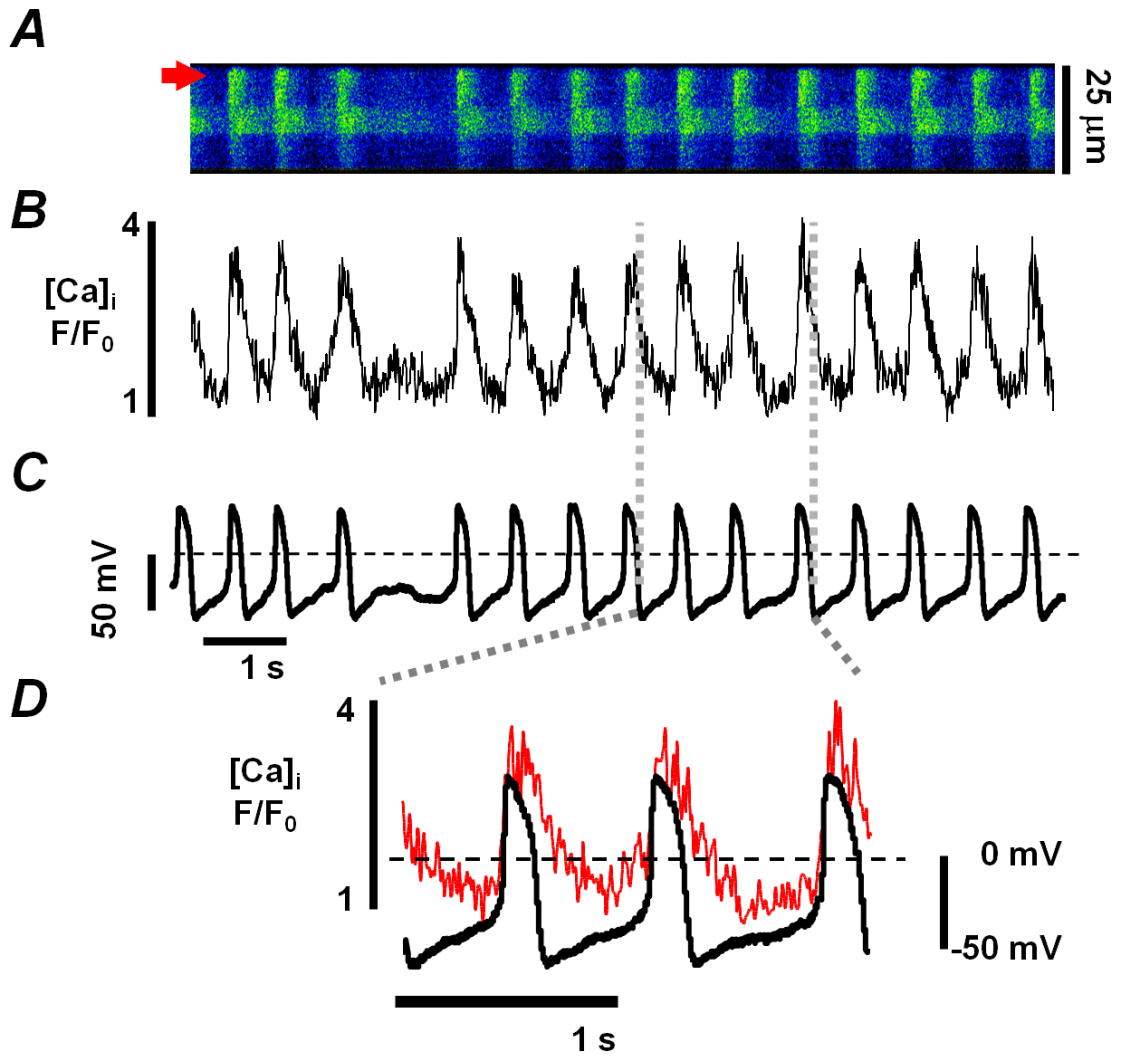
Interplay between spontaneous APs and [Ca^2+^]_i_ in ESdCs. Confocal line scan (**A**) and corresponding F/F_0_ plot (**B**) in a 17 day old ESdC with simultaneous measurement of changes in V_m_ (**C**). Spontaneous action potentials (APs) are recorded that correlate in time with Ca^2+^ transients. **D:** Superposition of Ca^2+^ transients and APs clearly show an increase in [Ca^2+^]_i_ in the late phase of the diastolic depolarization.

**Figure 2:**
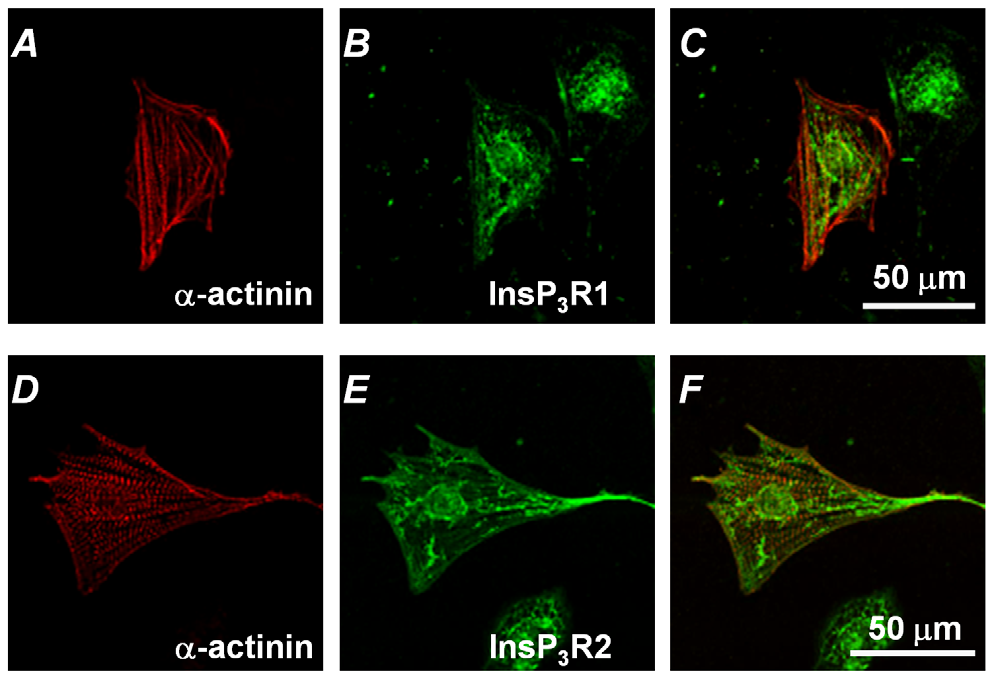
InsP3 receptor isoform expression in ESdCs. Dissociated ESdCs (d10–11) were stained for α-actinin (**A, D**), InsP_3_R1 (**B**) and InsP_3_R2 (**E**). **C,F:** Superposition of α-actinin and InsP_3_R staining.

In our previous study we demonstrated that InsP_3_-mediated Ca^2+^ release plays a critical role in the generation of spontaneous activity in ESdCs [9]. To determine whether the changes in [Ca^2+^]_i_ correlate with changes in membrane voltage (V_m_) we recorded action potentials (APs) in Fluo-4/AM loaded ESdCs with the perforated patch technique. As shown in Fig. 1, changes in [Ca^2+^]_i_ closely correlated with changes in V_m_ showing a clear increase in basal [Ca^2+^]_i_ during the diastolic depolarization. This increase in [Ca^2+^]_i_ was spatially homogeneous and did not correlate with a specific location inside the cell e.g. the nuclear envelope [26] or sub-sarcolemmal space [27]. To determine the location of InsP_3_Rs in ESdCs, cells were stained with antibodies against InsP_3_Rs type-1 and type-2. As shown in Fig. 2, ESdCs stained positive for both InsP_3_R isoforms. Pronounced peri-nuclear staining was identified, together with extensive endoplasmic reticulum staining throughout the cell that extended to the plasma membrane. This localization pattern suggests that InsP_3_R-mediated Ca^2+^ release is not restricted to the nuclear envelope.

**Figure 3:**
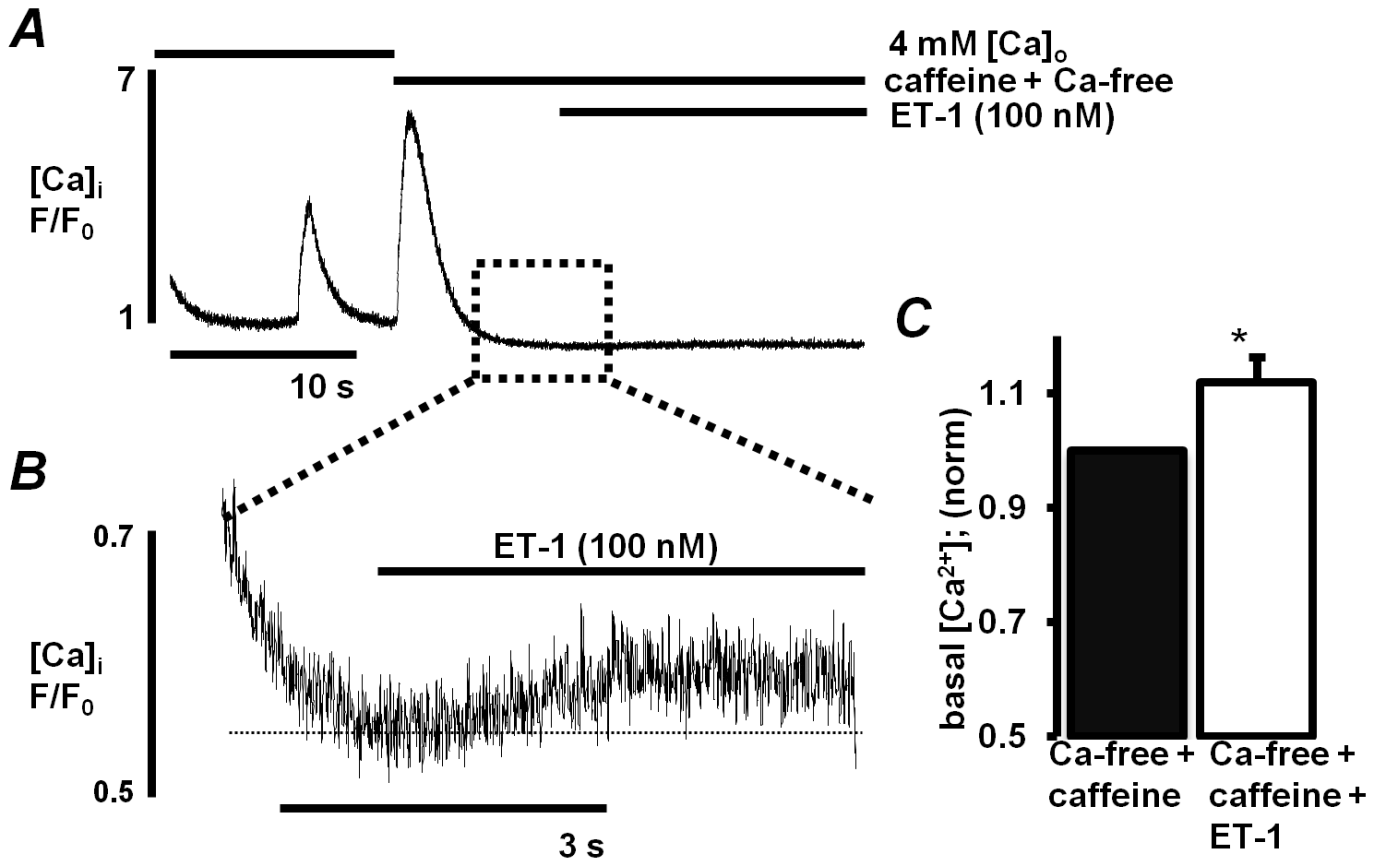
InsP_3_R controlled Ca^2+^ stores are in part functionally separated from RYR controlled stores. **A:** F/F_0_ plot of [Ca^2+^]_i_ in a spontaneously active ESdC after superfusion of the cell with tyrode solution supplemented with 4 mmol/L Ca^2+^. After depletion of ryanodine receptor operated Ca^2+^ stores by 10 mmol/L caffeine, ET-1 (100 nmol/L) induced an increase in basal [Ca^2+^]_i_. **B:** Magnification of the section of the F/F_0_ plot indicated by the box. **C:** Bar graph illustrating the increase in basal Ca^2+^ induced by ET-1 in the presence of caffeine (n = 6; *: P<0.05).

ESdCs express RyRs and caffeine induced Ca^2+^ transients have been recorded already when the cells first develop spontaneous activity [9], [28]. To evaluate whether RyRs and InsP_3_Rs control different functional pools of Ca^2+^ stored in the SR we superfused ESdCs with ET-1 (100 nmol/L) after the caffeine sensitive stores were depleted (caffeine: 10 mmol/L; Fig. 3A). The refilling of the stores was prevented by caffeine in the extracellular Ca^2+^-free solution. After recovery from the caffeine induced Ca^2+^ release, ET-1 induced a small but significant increase in basal [Ca^2+^]_i_ (Fig. 3BC). The ET-1 induced change indicates the presence of an InsP_3_R regulated SR Ca^2+^ pool in ESdCs that is independent of caffeine-sensitive stores. This is consistent with the fact that ESdCs maintain their spontaneous activity when RyR sensitive stores are depleted by caffeine [9].

**Figure 4:**
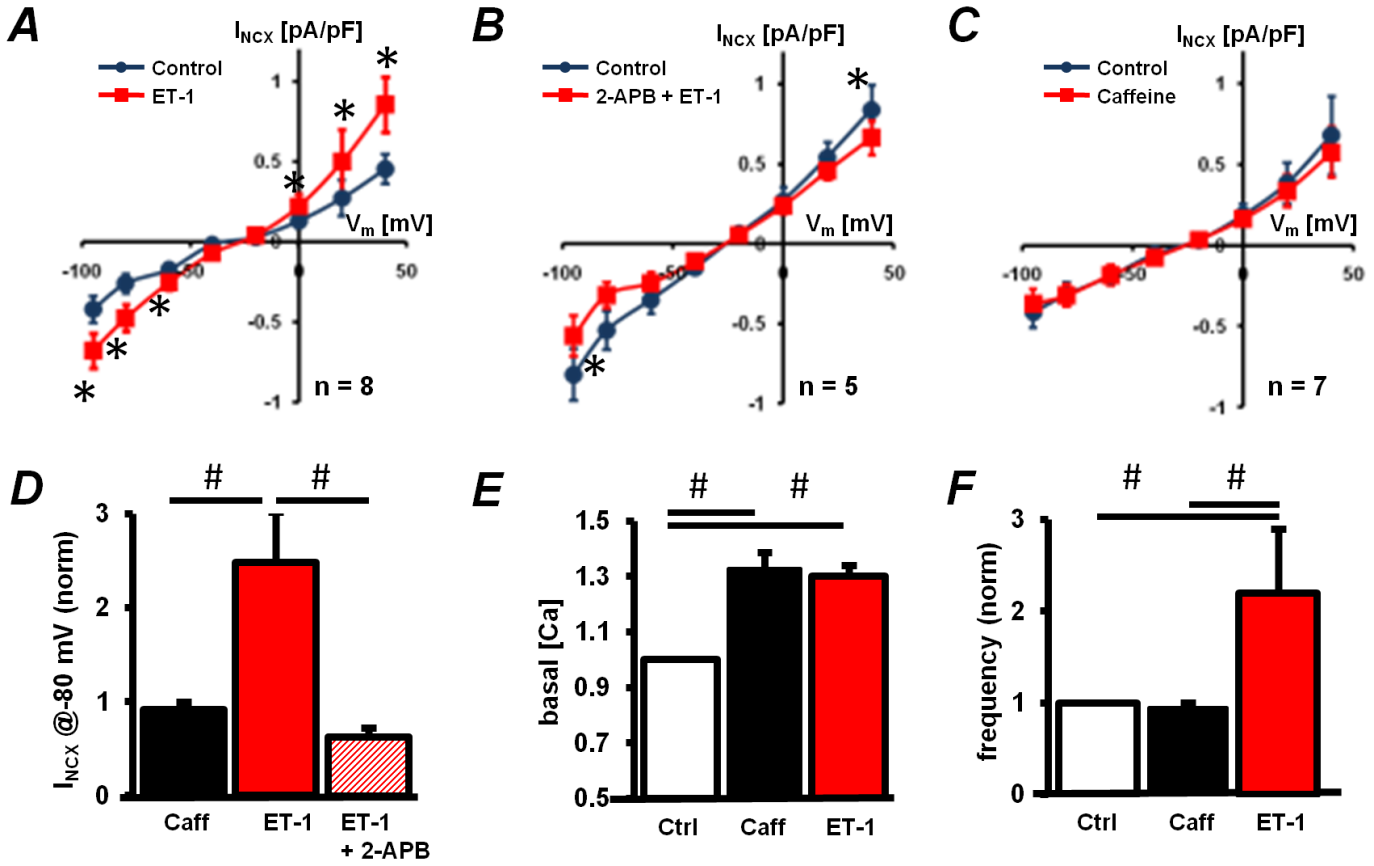
InsP_3_R induced Ca^2+^ release stimulates NCX activity. Current voltage plots for I_NCX_ recorded in ESdCs under control conditions and after 3 min superfusion with either **A:** ET-1 (100 nmol/L; n = 8), **B:** ET-1 (100 nmol/L)+2-APB (2 µmol/L; n = 5), or **C:** caffeine (Caff: 100 µmol/L; n = 7). Currents are corrected for the nickel (5 mmol/L) insensitive background. **D:** Normalized I_NCX_ recorded at −80 mV. I_NCX_ recorded on superfusion with ET-1 was significantly different from Caff and ET-1+2-APB. **E:** Normalized change in basal [Ca^2+^]_i_. Significant increase of basal [Ca^2+^]_i_ was observed upon superfusion with Caff and ET-1. **F:** ESdCs beating frequency after 3 min superfusion with ET-1 (n = 4) or caffeine (n = 6) respectively. *: paired t-test P<0.05; #: one way ANOVA P<0.05.

To determine if stimulation of InsP_3_Rs can influence NCX activity we measured I_NCX_ during the superfusion of ESdCs with ET-1 (100 nmol/L; for details on the voltage protocol see Data S1). A significant increase of I_NCX_ was determined in the presence of ET-1 (Fig. 4AD). This ET-1 induced increase, was inhibited by the InsP_3_R blocker 2-APB (2 µmol/L, Fig. 4BD). To determine whether the effect of ET-1 depended on an overall increase in basal [Ca^2+^]_i_ we measured I_NCX_ in ESdCs superfused with 100 µmol/L caffeine. At this concentration caffeine increases the open probability of RyRs and leads to increased diastolic [Ca^2+^]_i_. Caffeine and ET-1 both increased basal [Ca^2+^]_i_ to a similar extent (Fig. 4E). However, the frequency of spontaneous Ca^2+^-transients was only increased during ET-1 superfusion while it remained unchanged in the presence of caffeine (Fig. 4F). Consistent with this, I_NCX_ remained unchanged following 3 min of superfusion with caffeine (Fig. 4CD). These findings support that InsP_3_R dependent Ca^2+^ release more efficiently enhances I_NCX_ activity.

**Figure 5:**
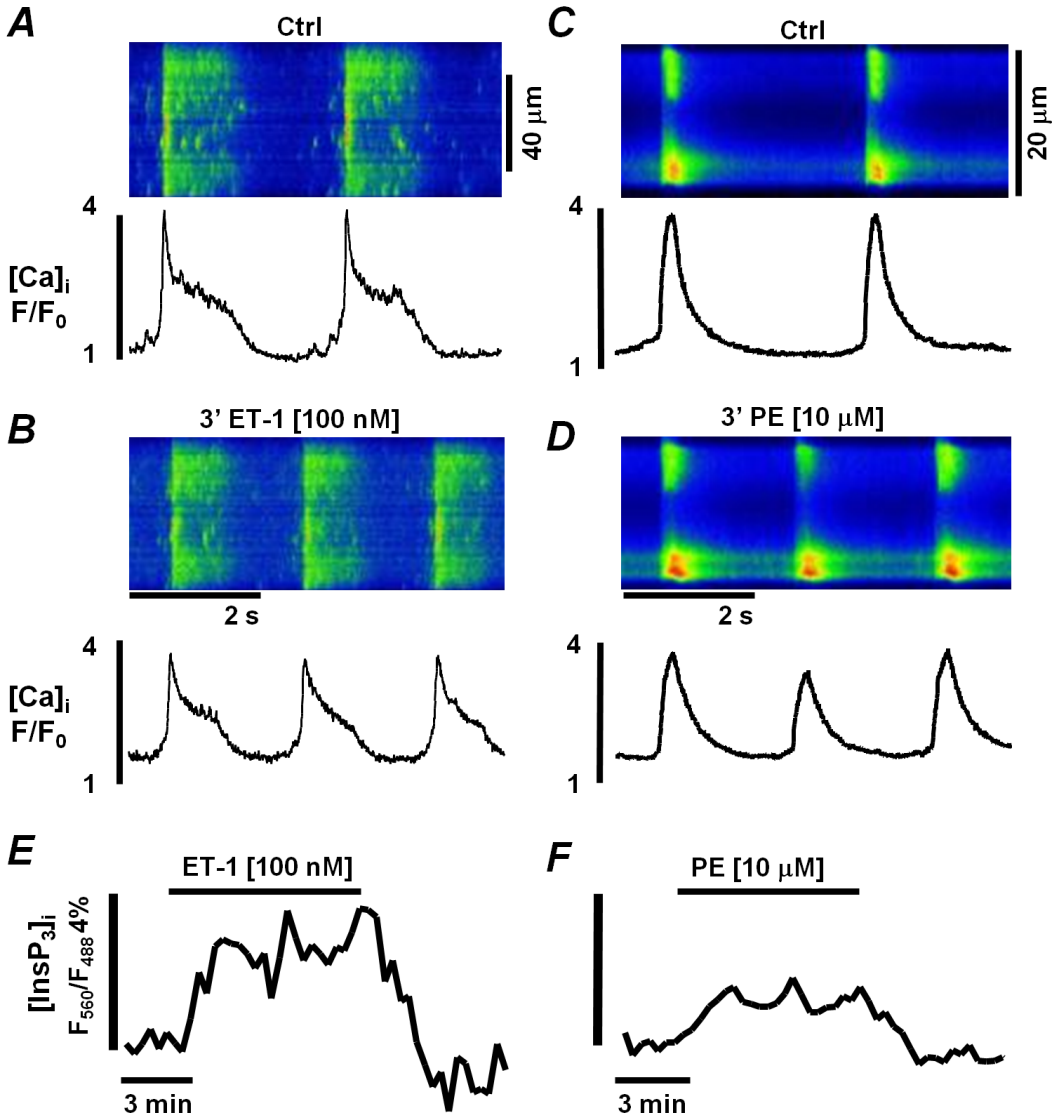
Agonist induced InsP_3_ increase regulates ESdC beating frequency. Line scan and corresponding F/F_0_ plots from spontaneously active ESdCs in Ctrl conditions (**A, C**) and after superfusion with either **B:** ET-1 (100 nmol/L) or **D:** PE (10 µmol/L). Superfusion of FIRE-1 expressing cells with **E:** ET-1 (n = 4) or **F:** PE (n = 2) induced an increase in the fluorescence ratio F560/F488 indicating an increase in [InsP_3_]_i_.

**Figure 6:**
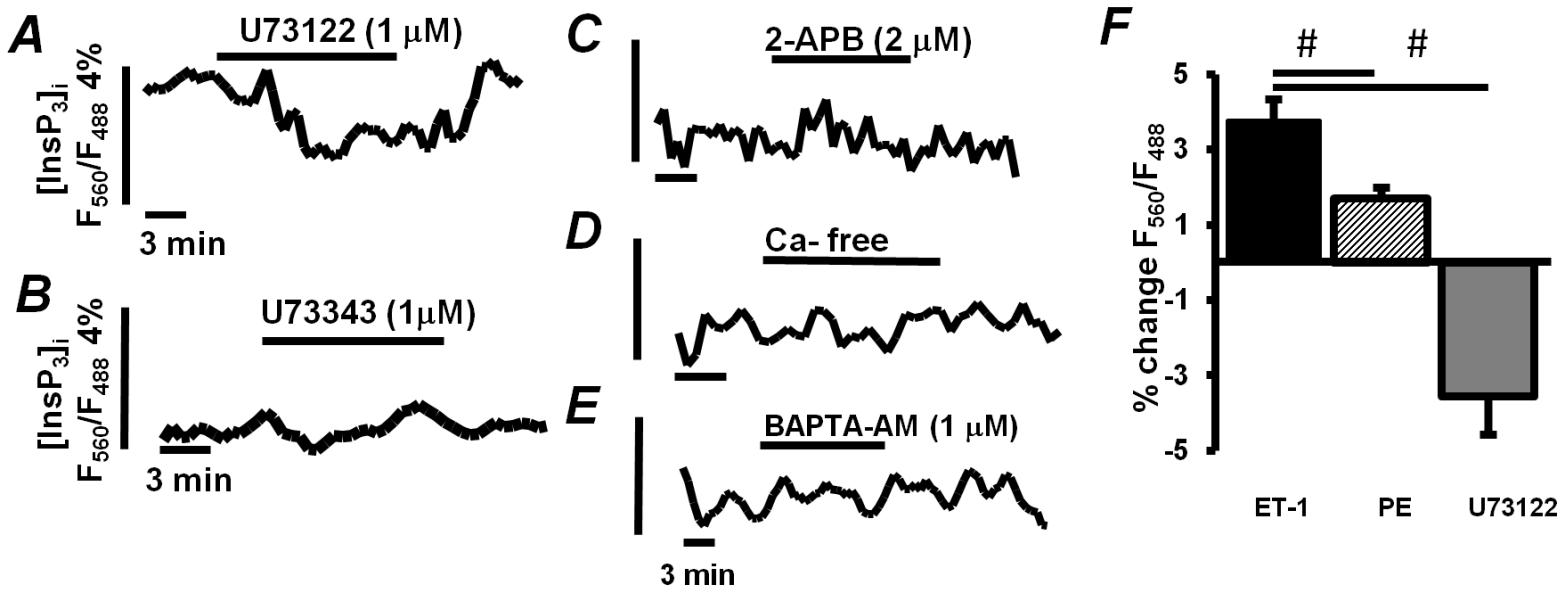
Changes in [IP_3_]_i_ are independent of [Ca]_i_. Fluorescence ratio F560/F488 measured in FIRE-1 expressing ESdCs superfused with **A:** the PLC inhibitor, U73122 (1 µmol/L; n = 5), **B:** its inactive analog, U73343 (1 µmol/L; n = 2), **C:** the InsP_3_R blocker, 2-APB (2 µmol/L; n = 2), **D:** Ca^2+^ -free solution (n = 2) or **E:** BAPTA-AM. **F:** The normalized changes of F_560_/F_488_ recorded in ET-1, PE or U73122. #: one way ANOVA P<0.05.

To determine the spatial organization of InsP_3_ signaling in ESdCs we transduced cells with an adenovirus expressing FIRE-1 (24 h). FIRE-1 exhibits an increase in the fluorescence ratio (F_560_/F_480_) upon binding of InsP_3_ [24]. When InsP_3_ production in ESdCs was stimulated by ET-1 (100 nmol/L) or PE (10 µmol/L) a positive chronotropic effect was determined in the frequency of the spontaneous Ca^2+^ transients (Fig. 5AB and CD, respectively) with a 3.0±1.1 fold (from 0.13±0.03 Hz to 0.32±0.06 Hz; n = 4) and a 1.5±0.3 (from 0.5±0.12 Hz to 0.65±0.1 Hz; n = 5) fold increase in the frequency after 3 minutes of superfusion, respectively. In FIRE-1 expressing ESdCs the same superfusion protocol was applied. When the fluorescence was integrated over the entire width of the cell, an ET-1 or PE induced increase in the FRET ratio (F_560_/F_488_) was determined that reached a steady state after about 2.5 min of superfusion. For ET-1, a 3.7±0.6% change (Fig. 5E & 6F; n = 4) in the FRET ratio was determined while the change for PE amounted to 1.7±0.03% (Fig. 5F & 6F; n = 2). The increase returned to baseline upon washout (Fig. 5E and F). When FIRE-1 infected ESdCs were superfused with the PLC inhibitor U73122 (1 µmol/L) a reversible decrease in F_560_/F_488_ (Fig. 6A; n = 5) was determined indicating a reduction in basal InsP_3_ production. U73343 (1 µmol/L) the inactive analog of U73122 remained without effect (Fig. 6B; n = 2). To exclude that changes in FRET ratio with U73122 were due to a decrease in basal [Ca^2+^]_i_ we used 3 alternative approaches to reduce basal [Ca^2+^]_i_ and spontaneous activity in ESdCs. During superfusion of ESdCs with either 2-APB (Fig. 6C), Ca^2+^-free solution (Fig. 6D) or BAPTA-AM (Fig. 6E) the florescent ratio F560/F488 was determined. We had previously demonstrated that these interventions attenuate ESdCs spontaneous activity and reduce basal [Ca^2+^]_i_ by 18.1±6.9% (n = 2) and 18.6±0.36% (n = 2), and 27.05±1.5% (n = 3), respectively [9]. Under none of the conditions, (Ca^2+^-free, n = 3; 2-APB: 2 µmol/L, n = 3; or BAPTA/AM: 1 µmol/L, n = 2) was a change in the FRET ratio (F560/F488) measured indicating that changes in FIRE-1 did not depend on changes in [Ca^2+^]_i_ or spontaneous activity.

FIRE-1 transduction changed the spontaneous activity in ESdCs. The overall number of spontaneously active cells was reduced and in beating cells the frequency of spontaneous Ca^2+^ transients was attenuated (FIRE-1: 0.43±0.09 Hz; n = 5) compared to non-transduced cells (Control: 0.93±0.1 Hz; n = 11) of the same age (day 16). The results indicate that the InsP_3_ buffer capacity of FIRE-1 in ESdCs reduces the beating frequency. To determine if frequency regulation through InsP_3_-mediated Ca^2+^-release depends on defined InsP_3_ signaling domains we employed two different strategies. First we limited peri-nuclear and nuclear InsP_3_ signaling by expression of FIRE-1 with a nuclear localization sequence (FIRE-1nuc), second we limited sub-sarcolemmal InsP_3_ signaling by over-expression of the membrane associated inositol polyphosphate 5-phosphatase m43 [29]. The enzyme m43 rapidly degrades InsP_3_ by removing the 5′ phosphate [25].

**Figure 7:**
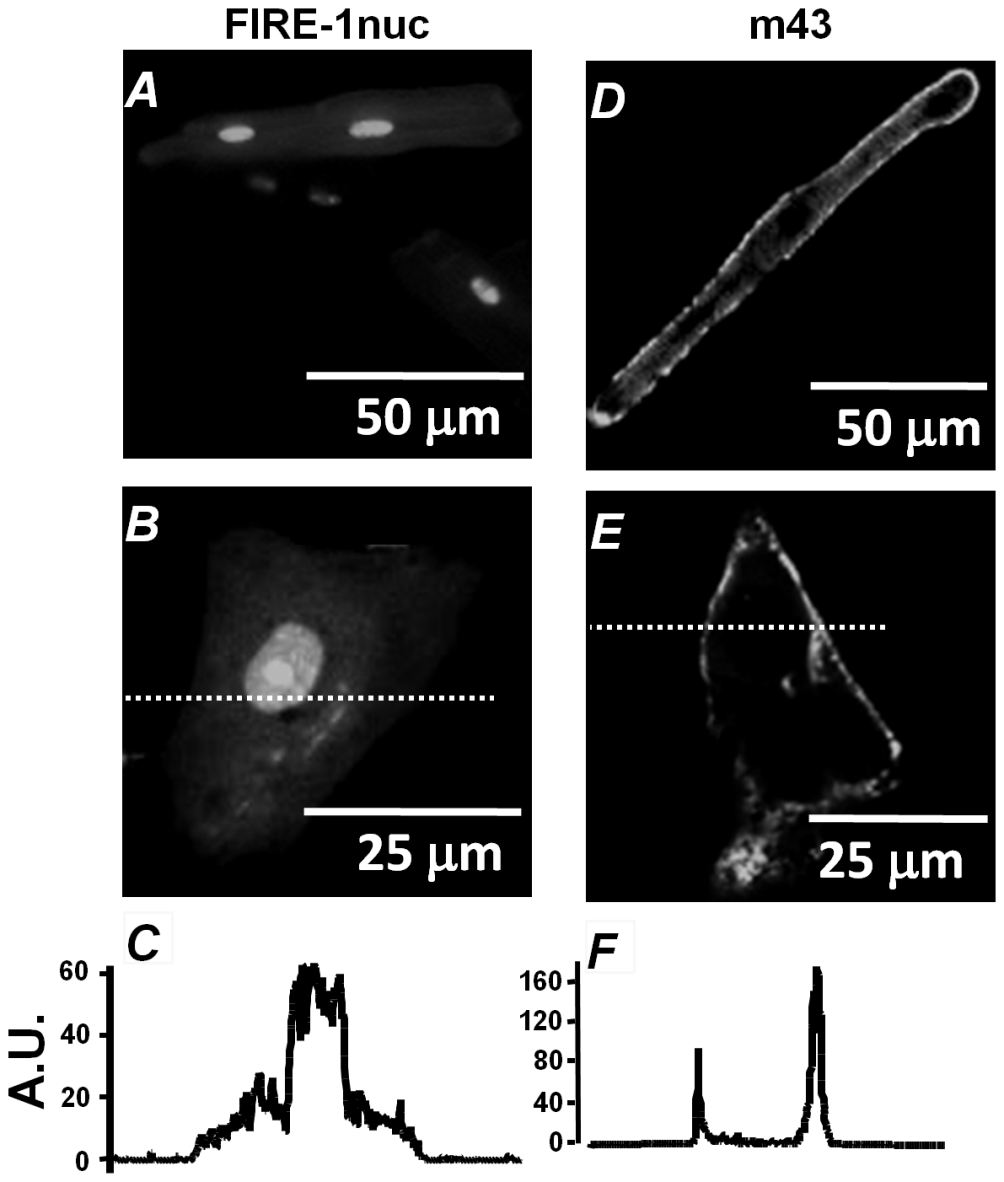
Subcellular buffering of [InsP_3_] at the nucleus and the plasma membrane. FIRE-1nuc infected **A:** ventricular myocytes and **B:** ESdCs exhibit pronounced YFP-fluorescence in the nucleus as demonstrated by the fluorescence plot (**C**) along the line shown in **B**. Immunostaining of m43 infected **D:** atrial myocyte and **E:** ESdCs with antibodies against FLAG tag. **F:** Fluorescence plot along the line shown in **E** demonstrates that m43 localizes predominantly to the plasma membrane.

The spatially defined localization of both FIRE-1nuc and m43 was confirmed by adenoviral transduction of the two constructs in atrial and ventricular myocytes as well as ESdCs. FIRE-1nuc was readily identified by its YFP fluorescence and the m43-phosphatase was visualized with an antibody against the incorporated FLAG-tag. Figure 7 shows a cat ventricular myocyte (A) and an isolated ESdC (B) expressing FIRE-1nuc. The fluorescence profile (C) obtained along a line positioned through the ESdC demonstrates the predominant localization of FIRE-1nuc to the nuclear envelope. The sub-cellular localization of m43 was determined through immunoblotting of fractionated whole cell lysate from COS-1 cells expressing FLAG-tagged m43 (SFig. 2) and immunostaining of transduced cat atrial myocytes (Fig. 7D) and ESdCs (Fig. 7E). Immunoblotting clearly localizes m43 in the membrane fraction of the cell lysate and immunostainings show a preferential localization of m43 at the plasma membrane of atrial myocytes and ESdCs 24 hours post adenoviral transduction. The distribution is in agreement with previous findings of Vasilevski et al. (2008) [25].

**Figure 8:**
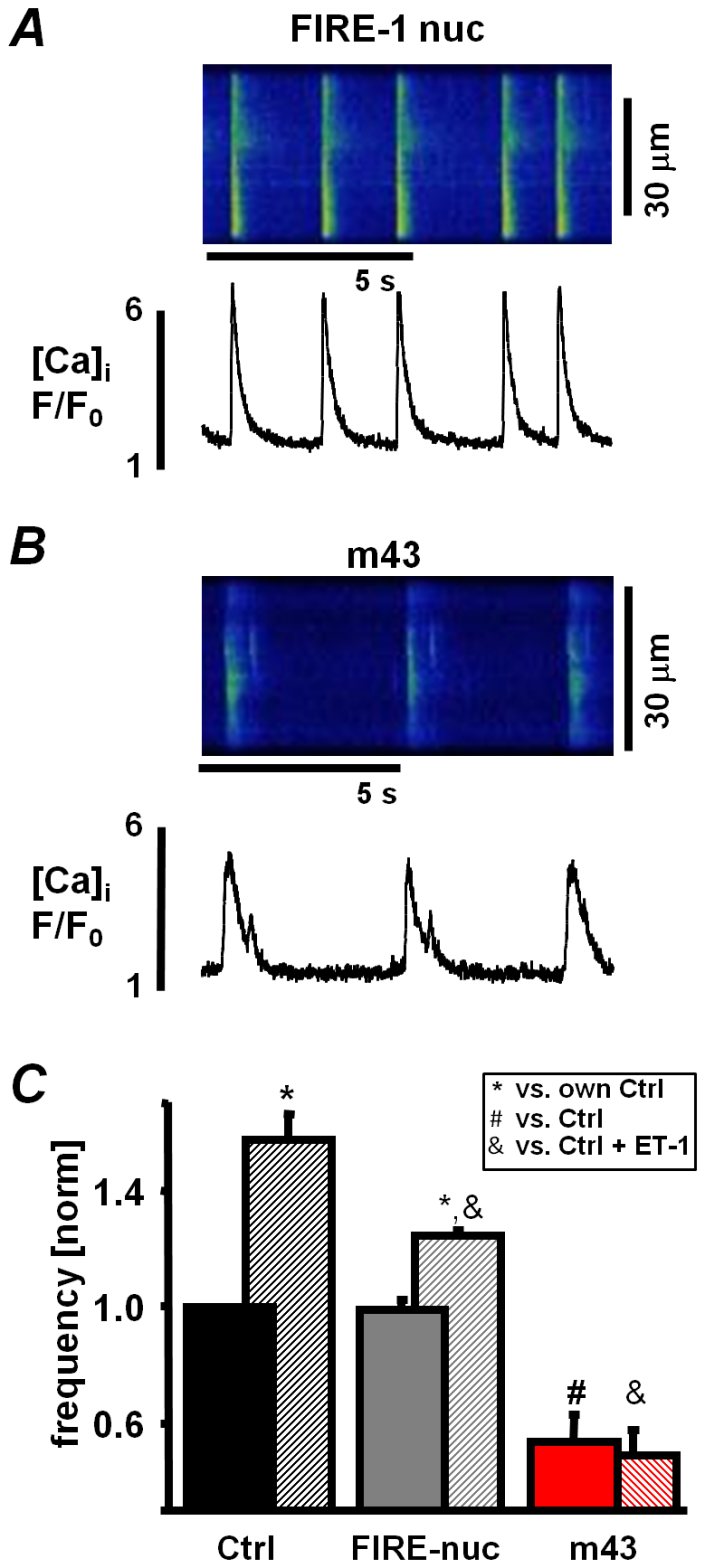
Pacemaker activity in ESdCs is regulated by sub-sarcolemmal Ca^2+^ release. Line scan and F/F_0_ plot from **A:** FIRE-1nuc and **B:** m43 infected spontaneously active ESdCs. **C:** Normalized beating frequency of control (black, n = 7), FIRE-1nuc (grey, n = 6) and m43 (red, n = 5) infected ESdCs in ctrl and after superfusion with ET-1 (hatched; n = 3, n = 4, n = 5, respectively). (*: P<0.05 compared to endogenous Ctrl; #, &: P<0.05 compared to Ctrl or Ctrl+ET-1, respectively).

To determine how localized suppression of InsP_3_ signaling effects the spontaneous activity of ESdCs we measured [Ca^2+^]_i_ in cells expressing m43 or FIRE-1nuc 24 hours post adenoviral transduction. Figure 8A shows spontaneous whole cell Ca^2+^ transients in an ESdC expressing FIRE-1nuc. Non-transduced 14 days old cells obtained from the same isolation served as control. Control ESdCs and ESdCs transduced with FIRE-1nuc exhibited no significant difference in their Ca^2+^ transient frequency (0.51±0.05 Hz; n = 7 and 0.44±0.02 Hz; n = 6, respectively; Fig. 8C). Upon stimulation with ET-1 the frequency of spontaneous Ca^2+^ transients increased 58±9% (n = 3) in control and 24±1% (n = 4; *P*<0.05) in FIRE-1nuc transduced ESdCs. The data indicate that the ET-1 induced positive chronotropic effect persists when InsP_3_R is buffered in the nucleus of ESdCs. In contrast, cells transduced with m43 exhibited a significantly reduced beating frequency in comparison to control and FIRE-1nuc cells (54±9% of control; n = 5; *P*<0.05; Fig. 8BC) and an ET-1 induced positive chronotropic effect was not observed (Fig. 8C). The data support the hypothesis that membrane delineated inhibition of InsP_3_ signaling can efficiently modulate the spontaneous activity of ESdCs.

## Discussion

In the present study we demonstrate that a basal production of InsP_3_ maintains spontaneous activity in ESdCs by regulating Ca^2+^-release from a SR Ca^2+^ pool that is functionally independent from RyR-mediated Ca^2+^-release. In addition we show that while InsP_3_ production changes [Ca^2+^]_i_ throughout the cytoplasm, the InsP_3_R signaling domains relevant for NCX activation and spontaneous activity are localized close to the plasma membrane where their Ca^2+^ release is efficiently translated into a depolarization of the membrane potential.

### InsP_3_Rs in developing cardiomyocytes

All three InsP_3_R subtypes -1, -2, and -3 are expressed in un-differentiated ES cells [30] and embryonic cardiomyocytes [1], [3], [31] where InsP_3_R1 is most prevalent in the nuclear envelope [3], [10]. We have identified InsP_3_R1 and InsP_3_R2 in ESdCs (see Fig. 2B,E) with a sub-cellular distribution comparable to that in neonatal myocytes [4]. Previous studies also suggested that InsP_3_R1 maintains spontaneous activity in embryonic cardiomyocytes which was suppressed with introduction of antisense cDNA of InsP_3_R1 [10].

### Ca^2+^ release from the sarcoplasmic reticulum by InsP_3_Rs

The SR is a continuous network [32] where Ca^2+^ can redistribute [18], [33]–[35]. InsP_3_Rs and RyRs localize to and deplete the same SR network in rabbit ventricular myocytes [33]. Interestingly our data demonstrate that InsP_3_R-mediated Ca^2+^ release can still be induced when RyR-controlled Ca^2+^ stores were depleted by caffeine. This supports the hypothesis that InsP_3_R signaling domains are functionally isolated and not immediately affected by RyR-controlled Ca^2+^ store depletion. A similar finding was described in colonic smooth muscle cells where in an interconnected SR network, Ca^2+^ release from RyR or InsP_3_R controlled stores could be demonstrated after depletion of the respective other InsP_3_ or caffeine sensitive store [36]. In ESdCs the size of this functionally independent InsP_3_ sensitive Ca^2+^ store remains to be determined but as demonstrated, it is sufficient to maintain spontaneous activity of ESdCs [9].

### Role of InsP_3_Rs for the generation of spontaneous activity

We and others have demonstrated that Ca^2+^ release plays a dominant role in the generation of spontaneous Ca^2+^ transients in mouse [3], [7], [9], [10], [37] and human embryonic cardiomyocytes [11], [26]. The transients coincide with changes in V_m_, and the late phase of the diastolic depolarization is accompanied by an increase in [Ca^2+^]_i_ (Fig. 1). A similar increase in [Ca^2+^]_i_ was described in cat latent pacemaker cells and cat and rabbit sinus nodal cells [27], [38] where sub-sarcolemmal Ca^2+^ release from RyRs is proposed to enhance a depolarization of V_m_ by activation of NCX.

In latent pacemaker and sinus nodal cells the Ca^2+^ release events that drive the depolarization are localized in the sub-sarcolemmal space. However, in ESdCs it was proposed that the Ca^2+^ release that initiates the diastolic depolarization originates at the nuclear envelope [7], [8], . This was supported by the nuclear localization of InsP_3_Rs and the demonstration of peri-nuclear InsP_3_R-mediated Ca^2+^ release [7], [8],[40]. While most of the InsP_3_ synthesis occurs at the plasma membrane PLCs and InsP_3_ production are also described within the nuclear envelope [41]. In our experiments nuclear InsP_3_ buffering through FIRE-1nuc had no significant effect on Ca^2+^ transient frequency thus excluding a major contribution of nuclear PLCs to spontaneous activity.

In cardiac myocytes, stimulation of InsP_3_R-mediated Ca^2+^ release by ET-1 can induce spontaneous arrhythmic Ca^2+^ transients although RyRs outnumber InsP_3_Rs by 50∶1 [19], [20], [42]. Differences in InsP_3_R to RyR signaling are also reflected in our data where ET-1 but not caffeine has a positive chronotropic effect (Fig. 4F) in ESdCs. The efficient translation of InsP_3_-mediated Ca^2+^ release into a depolarization of V_m_, could depend on the localization of InsP_3_R close to the plasma membrane or within a functional signaling domain. A close apposition was demonstrated in rat atrial myocytes [43], and proposed in rat ventricular myocytes [19]. Data from Harzheim et al. (2009) [20] show that in hypertrophic rat ventricular myocytes InsP_3_Rs predominantly increase in the cytoplasm and correlate with enhanced ET-1 induced arrhythmic activity. Our data demonstrate that over-expression of the InsP_3_ 5-phosphatase m43 [25], [29] in the plasma-membrane [44] decreased ESdCs beating frequency and abolished ET-1 induced positive chronotropy (Fig. 8C). This is consistent with previous results from neonatal cardiomyocytes where m43 reduced the InsP_3_ response after α-adrenergic stimulation [25] and supports that the InsP_3_ production and the InsP_3_R-mediated Ca^2+^ release relevant to spontaneous activity occurs at the plasma membrane.

In addition to a preferred sub-sarcolemmal location of InsP_3_Rs, the formation of a specialized signaling domain could explain the efficient translation of Ca^2+^ release into changes of V_m_. Signaling domains between InsP_3_Rs and the effector proteins NCX or the Ca^2+^ activated chloride channel have been demonstrated. The adaptor protein ankyrin [45] that binds to NCX and InsP_3_R [46] could form a potential linker that maintains a close spatial and functional proximity between the proteins. Recent data show that decreased levels of ankyrin attenuate sinus node activity [47], [48]; so future experiments will have to reveal how ankyrin loss changes the functional coupling between InsP_3_R mediated Ca^2+^ release and I_NCX_.

## Conclusion

In the current study we demonstrate that spontaneous activity in ESdCs depends on sub-sarcolemmal signaling domains of InsP_3_R and NCX that allow an efficient translation of InsP_3_R-mediated Ca^2+^ release into a depolarization of the plasma membrane. While the InsP_3_ signaling domain described around the nucleus of adult and neonatal ventricular myocytes might enable excitation-transcription coupling, sub-sarcolemmal InsP_3_ signaling has significant impact on cellular excitability and arrhythmicity. The data indicate that pathological changes in cardiac muscle cells might not only depend on the level of InsP_3_R expression but more critically on their location within the myocytes.

## Supporting Information

Data S1 **Supporting information.**
(DOC)

Figure S1 **A.** Fluorescent images of an ESdC taken at >560 nm (top) and 488 nm (bottom) before (left) and after bleaching (right). **B.** Bar graphs display the change in CFP (right) and decrease of YFP (right) fluorescence after photobleaching (hatched bar, n = 3). The results are comparable to bleaching experiments in FIRE-1 expressing COS-1 cells [12].
(TIF)

Figure S2 **Western blot of the cytoplasmic (soluble) and membrane fraction (pellet) of M43 transfected COS cells.** Blots probed with the anti-tag antibody show positive M43 immunostaining only in the membrane fraction.
(TIF)
