# Supplementary material for: Spatially Defined InsP3-Mediated Signaling in Embryonic Stem Cell-Derived Cardiomyocytes
Source: PLoS One. 2014 Jan 7;9(1):e83715. doi: 10.1371/journal.pone.0083715 (PMC3883750; doi:10.1371/journal.pone.0083715)
Supplement: Data S1 — Supporting information. (DOC) [file pone.0083715.s001.doc]

**Supplemental Data**

**Methods**

*Culture and differentiation of mouse embryonic stem cells*

Mouse ES cells (CMV, Specialty Media; Phillipsburg, NJ) transfected with a neomycin resistance gene expressed under the promoter of αMHC [2,3] were propagated on inactivated neomycin resistant feeder layers (Specialty Media; Phillipsburg, NJ) in the presence of Leukemia Inhibiting factor (LIF: ESGRO; 1000 U/ml; Chemicon Int., Temecula, CA). For differentiation embryoid bodies (EBs) were prepared [4,5,6] that were plated after day 7. Spontaneous beating of cardiomyocytes could be observed within a day of plating [5]. At that point neomycin (300 μg/ml) was added to the EBs to select for cardiomyocytes. For dissociation cardiomyocyte aggregates were collected and washed in a Ca^2+^- and Mg^2+^-free phosphate buffered saline solution (PBS, Sigma-Aldrich, St. Louis, MO). Sixty minute incubation in collagenase (1mg/ml at 37^o^C; Sigma-Aldrich, St. Louis, MO) and KB solution ([7] 60 min; room temperature) resulted in a single cell suspension. These cells were plated onto gelatin coated coverslips in Iscove media supplemented with 20% FBS (Invitrogen, Frederick, MD).

*Intracellular Ca^2+^ measurements*

The intracellular Ca^2+^ concentration of single ESdCs was monitored with the fluo-4 acetoxymethyl ester (5 µM; fluo-4/AM; Invitrogen, Eugene, OR, USA). Cells were incubated (15 min) at room temperature and de-esterification of the dye was allowed for 15 min. Whole-cell Ca^2+^-transients were obtained from confocal linescan images (4ms/line; Excitation 488 nm, emission: >512 nm) through single ESdCs. Ca^2+^-transients are presented as background-subtracted normalized fluorescence (F/F_0_) where the fluorescent was averaged over the entire width of the cell, unless indicated otherwise. During the experiments cells were superfused with a tyrode solution containing (mmol/L): NaCl 140; KCl 5.4; CaCl_2_ 1.5; MgCl_2_ 1.5; glucose 10; Hepes 5 (pH adjusted to 7.38 with NaOH).

*Patch Clamp Recordings I_NCX_*

For I_NCX_ measurements [Ca^2+^]_i_ was buffered to 150 nmol/L (calculated by using the Maxchelator program, D. Bers, SU Davis, Davis, CA). The external solution contained (in mM): CsCl 10, NaCl 135, CaCl_2_ 2, MgCl_2_ 1, Glucose 10, Hepes 10, niflumic acid 0.1, Ouabain 0.01 and verapamil 0.01, adjusted to pH 7.4 (CsOH). The pipette solution contained (in mmol/L): CsCl 136, NaCl 10, Aspartic acid 42, MgCl 3, Hepes 5, TEA 20, MgATP 10 and 150 nmol/L free [Ca^2+^]_i_, adjusted to pH 7.4 (CsOH). ESdCs membrane potential was held at –30 mV. I_NCX_ was measured by application of a slow-voltage ramp protocol (+60 mV to –100 mV) at 90 mV/s that was run in 10 s intervals. To distinguish I_NCX_ from potential leak currents all cells were superfused with extracellular tyrode supplemented with Ni^2+^ (5 mM) and I_NCX_ is displayed as the I_NCX_ = (I_total_-I_Ni_). Experiments were performed at room temperature with patch electrodes prepared from borosilicat glass (WPI) with a tip resistance of 3 to 5 MΩ.

*Simultaneous measurements of [Ca*^2+^*]_i_ and action potentials*

Current clamp recordings were performed in the perforated-patch technique. The pipette solution contained (in mmol/L): KAsp 100; KCl 15; KH_2_PO4 5; MgATP 5; EGTA 0.4; CaCl_2_ 0.12; MgCl_2_ 0.75; phosphocreatine 10; HEPES 10; pH 7.2 adjusted with KOH. Amphotericin B stock solution (3 mg/50 μl DMSO) was added to the pipette solution at a final concentration of 240 μg/ml. The final solution was maintained on ice and used within 3 hours of preparation. Spontaneous electrical activity was recorded in the current clamp mode using an Axopatch 200A patch-clamp amplifier (Axon Instruments, Union City, CA, USA) and PCLAMP6 software (Axon Instruments).

## *Immunocytochemistry*

ESdCs plated on gelatin coated coverslips (12 mm diameter) were fixed with 4% paraformaldehyde (30 min; 37^o^C) and stained as previously described [8]. Triton permeabilized cells (Triton X-100: 0.3%, 30 min; Sigma) were incubated with primary antibodies i) T1NH (InsP_3_R type-1 amino-terminal antibody; 1:100); ii) T2NH (InsP_3_R type-2 amino-terminal antibody; 1:100); iii) T3NH (InsP_3_R type-3 amino-terminal antibody; 1:100) [9]; iv) antibodies against the FLAG epitope (DYKDDDDK; Affinity BioReagents, CO, USA) (1:1000), or v) against α-actinin (1:1600; Sigma A7811) overnight at 4^o^C. Incubation with the secondary antibodies (Texas Red 1:500; goat anti mouse, Molecular Probes T862) for α-actinin, and Alexa Fluor 488 (1:200; goat anti rabbit, Invitrogen A11008) for the remaining primary antibodies) was maintained for 2 h at room temperature. Cells were mounted onto microscope slides with the mounting medium Fluoromount-G (Southern Biotech; Birmingham, AL, US). The immunostainings were analyzed by confocal microscopy.

*COS-1 Cell Transfection and Infection*

COS-1 cells were transiently transfected with the expression plasmid for m43 using a diethylaminoethyl-dextran method as previously described [10]. COS-1 cells used for adenoviral infection were cultured in serum-free Dulbecco’s modified Eagle’s medium and exposed to m43 adenovirus fro 2hr. at a multiplicity of infection 1-10. The COS-1 cells were subsequently cultured for 24-36hr. before harvesting.

*Cellular Fractionation*

COS-1 cells were harvested in lysis buffer (50mmol/liter Tris-HCl pH 8.3, 1 mmol/L EDTA, 5 mmol/liter sodium azide, 0.25 mmol/L PMSF, 10 µmol/liter leupeptin, 10µmol/liter pepstatin, 100 µg/ml trypsin inhibitor) and lysed by passage through a 27-gauge syringe. Lysates were subjected to centrifugation for 10 min at 112,000 × g_max_. The supernatant containing cytosolic protein was used as the soluble fraction, while the pellet was used as the membranous fraction. Protein concentration was determined using BCA (Pierce).

*SDS-Polyacylamide Gel Electrophoresis and Immunoblotting*

SDS-PAGE and Western blotting were performed as described previously [11] using 7.5% SDS-polyacrylamide gels. Proteins were transferred to nitrocellulose membrane and immunoblotted using α-FLAG antibody (Affinity BioReagents). Visualization was accomplished using ECL reagents (Amersham Biosciences).

**Supplemental Results**

*FIRE-1*

To verify FRET between CFP and YFP in the FIRE-1 construct, we conducted a control experiment where the increase in donor (CFP) fluorescence was monitored upon bleaching of the acceptor (YFP). YFP was bleached by exposure of FIRE-1 expressing ESdC to the excitation wavelength of YFP (514 nm) at 100% intensity for 3 minutes. Fluorescent images of the cells were taken at the emission wavelength of CFP (488 nm) and the emission wavelength of YFP (>560 nm) before and after bleaching. A 14.9 ± 2 % increase of CFP fluorescence and a 44.7 ± 11 % decrease of YFP fluorescence upon photobleaching was determined (Fig. S1, n = 3), indicating that FRET is suppressed after acceptor bleaching. The results are comparable to bleaching experiments in FIRE-1 expressing COS-1 cells [12].

*Subcellular distribution of M43*

As demonstrated in Figure S2 protein isolated from the cytoplasmic fraction and from the membrane fraction of M43 transfected COS cells was subjected to western blotting. Concomitantly blots were subjected to immunostaining with the anti-tag antibody. Positive immunostaining is only detected in the pellet fractions further supporting the presence of M43 in the plasma membrane.

**References**

[1] Sheehan KA, Blatter LA. Regulation of junctional and non-junctional sarcoplasmic reticulum calcium release in excitation-contraction coupling in cat atrial myocytes. J Physiol 2003; 546: 119-32.

[2] Klug MG, Soonpaa MH, Koh GY, Field LJ. Genetically selected cardiomyocytes from differentiating embronic stem cells form stable intracardiac grafts. J Clin Invest 1996; 98: 216-24.

[3] Strom TB, Field LJ, Ruediger M. Allogeneic stem cells, clinical transplantation and the origins of regenerative medicine. Curr Opin Immunol 2002; 14: 601-05.

[4] Nagy A, Rossant J, Nagy R, Abramow-Newerly W, Roder JC. Derivation of completely cell culture-derived mice from early-passage embryonic stem cells. Proc Natl Acad Sci 1993; 90: 8424-8.

[5] Banach K, Halbach MD, Hu P, Hescheler J, Egert U. Development of electrical activity in cardiac myocyte aggregates derived from mouse embryonic stem cells. Am J Physiol Heart Circ Physiol 2003; 284: H2114-H23.

[6] Hescheler J, Wartenberg M, Fleischmann BK, Banach K, Acker H, Sauer H. Embryonic stem cells as a model for the physiological analysis of the cardiovascular system. Methods Mol Biol 2002; 185: 169-87.

[7] Klockner U, Lee JH, Cribbs LL, Daud A, Hescheler J, Pereverzev A, Perez-Reyes E, Schneider T. Comparison of the Ca2 + currents induced by expression of three cloned alpha1 subunits, alpha1G, alpha1H and alpha1I, of low-voltage-activated T-type Ca2 + channels. Eur J Neurosci 1999; 11: 4171-8.

[8] Fahrenbach JP, Mejia-Alvarez R, Banach K. The relevance of non-excitable cells for cardiac pacemaker function. J Physiol 2007; 585: 565-78.

[9] Ramos-Franco J, Caenepeel S, Fill M, Mignery G. Single channel function of recombinant type-1 inositol 1,4,5-trisphosphate receptor ligand binding domain splice variants. Biophys J 1998; 75: 2783-93.

[10] Mignery GA, Newton CL, Archer BT, 3rd, Sudhof TC. Structure and expression of the rat inositol 1,4,5-trisphosphate receptor. J Biol Chem 1990; 265: 12679-85.

[11] Mignery GA, Newton CL, Archer BT, Sudhof TC. Structure and expression of the rat inositol 1,4,5-trisphosphate receptor. J Biol Chem 1990; 265: 12679-85.

[12] Remus TP, Zima AV, Bossuyt J, Bare DJ, Martin JL, Blatter LA, Bers DM, Mignery GA. Biosensors to measure inositol 1,4,5-trisphosphate concentration in living cells with spatiotemporal resolution. J Biol Chem 2006; 281: 608-16.

**Supporting Information Legend**

**Figure S1:** **A.** Fluorescent images of an ESdC taken at >560 nm (top) and 488 nm (bottom) before (left) and after bleaching (right). **B.** Bar graphs display the change in CFP (right) and decrease of YFP (right) fluorescence after photobleaching (hatched bar, n = 3). The results are comparable to bleaching experiments in FIRE-1 expressing COS-1 cells [12].

**Figure S2:** Western blot of the cytoplasmic (soluble) and membrane fraction (pellet) of M43 transfected COS cells. Blots probed with the anti-tag antibody show positive M43 immunostaining only in the membrane fraction.
