# Supplementary figures and images for: Spatially Defined InsP3-Mediated Signaling in Embryonic Stem Cell-Derived Cardiomyocytes
Source: PLoS One. 2014 Jan 7;9(1):e83715. doi: 10.1371/journal.pone.0083715 (PMC3883750; doi:10.1371/journal.pone.0083715)

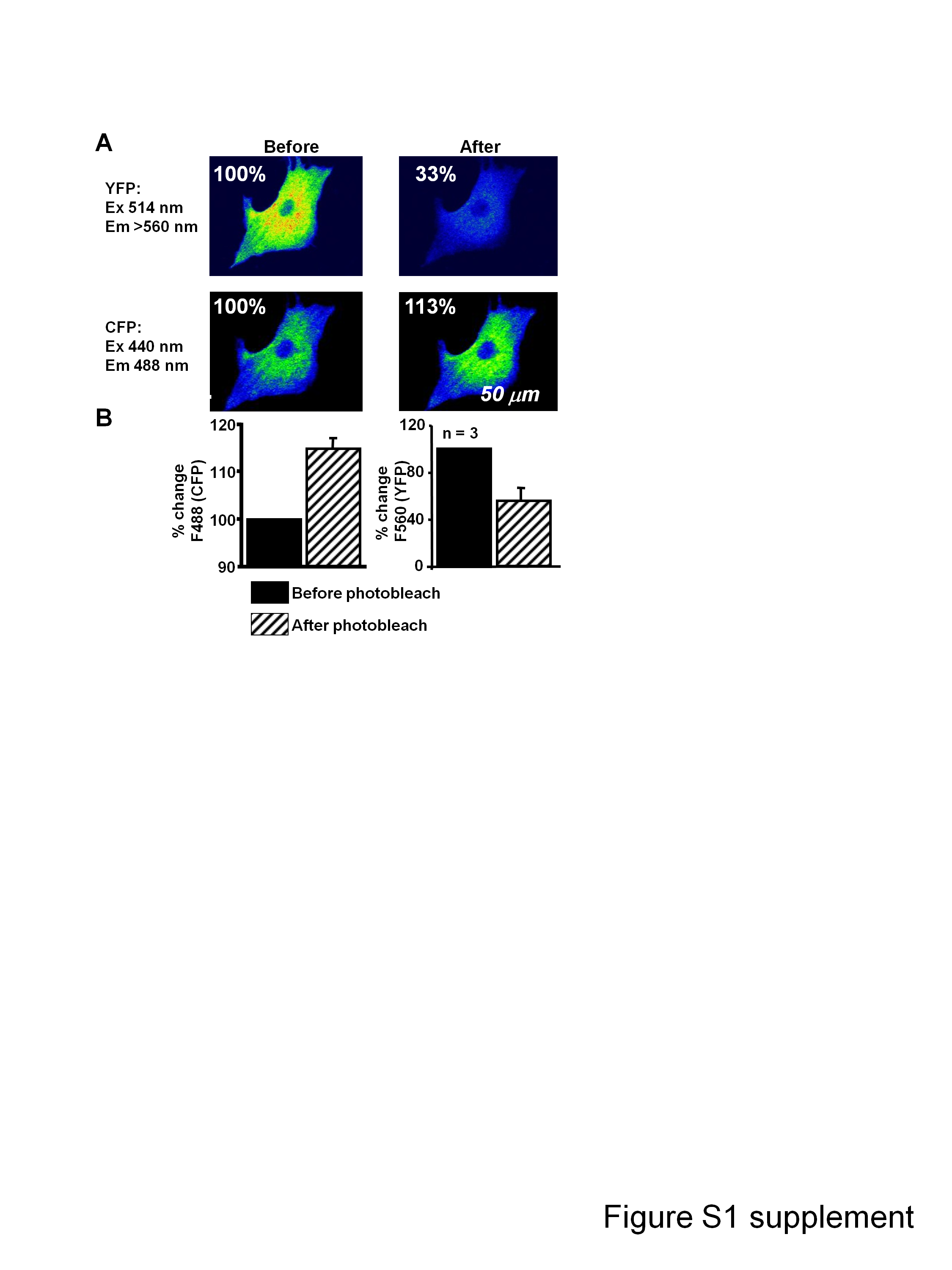

Supplement: Figure S1 — A. Fluorescent images of an ESdC taken at >560 nm (top) and 488 nm (bottom) before (left) and after bleaching (right). B. Bar graphs display the change in CFP (right) and decrease of YFP (right) fluorescence after photobleaching (hatched bar, n = 3). The results are comparable to bleaching experiments in FIRE-1 expressing COS-1 cells [12]. (TIF) [file pone.0083715.s002.tif]
